# Expression of the *Helicobacter pylori* virulence factor vacuolating cytotoxin A (*vac* *A*) is influenced by a potential stem‐loop structure in the 5′ untranslated region of the transcript

**DOI:** 10.1111/mmi.13160

**Published:** 2015-09-10

**Authors:** Karin R. Amilon, Darren P. Letley, Jody A. Winter, Karen Robinson, John C. Atherton

**Affiliations:** ^1^ Nottingham Digestive Diseases Biomedical Research UnitSchool of MedicineUniversity of NottinghamNottinghamUK; ^2^ Present address: The Roslin Institute and Royal (Dick) School of Veterinary StudiesUniversity of EdinburghEdinburghUK; ^3^ Present address: Pathogen Research GroupSchool of Science and Technology (Biosciences)Nottingham Trent UniversityNottinghamUK

## Abstract

The vacuolating cytotoxin, VacA, is an important virulence factor secreted by the gastric pathogen *H* *elicobacter pylori*. Certain *vac* *A* genotypes are strongly associated with disease risk, but the association is not absolute. The factors determining *vac* *A* gene expression are not fully understood, and the mechanisms of its regulation are elusive. We have identified a potential mRNA stem‐loop forming structure in the 5′ untranslated region (UTR) of the *vac* *A* transcript. Using site‐directed mutagenesis, we found that disruption of the stem‐loop structure reduced steady‐state mRNA levels between two‐ and sixfold (*P* = 0.0005) and decreased mRNA half‐life compared with wild type (*P* = 0.03). This led to a marked reduction in VacA protein levels and overall toxin activity. Additionally, during stressful environmental conditions of acid pH or high environmental salt concentrations, when general transcription of *vac* *A* was decreased or increased respectively, the stabilising effects of the stem‐loop were even more pronounced. Our results suggest that the stem‐loop structure in the *vac* *A* 5′ UTR is an important determinant of *vac* *A* expression through stabilisation of the *vac* *A* mRNA transcript and that the stabilising effect is of particular importance during conditions of environmental stress.

## Introduction

The Gram‐negative microaerophilic bacterium *Helicobacter pylori* persistently colonises the stomachs of, on average, half the world's population, causing chronic gastritis. In some individuals, this may eventually lead to the development of diseases such as gastric and duodenal ulcers, gastric adenocarcinoma and mucosa‐associated lymphoid tissue lymphoma (Atherton and Blaser, 2009). Disease risk is dependent on a combination of factors including the virulence of the infecting *H. pylori* strain, host genetics (El‐Omar *et al*., 2000) and environmental factors, such as diet (Wang *et al*., 2009) and smoking (Kurata and Nogawa, 1997).

The secreted vacuolating cytotoxin (VacA) is a major virulence factor, active forms of which are associated with increased risk of disease development (Atherton and Blaser, 2009). VacA is a pore‐forming toxin that displays a range of effects on epithelial cells *in vitro*, including vacuolation (Cover and Blaser, 1992), mitochondrial damage and apoptosis (Galmiche *et al*., 2000; Yamasaki *et al*., 2006). It also has immunomodulatory effects through inhibition of T‐cell activation and proliferation (Gebert *et al*., 2003; Torres *et al*., 2007).

The *vacA* gene is naturally polymorphic and can be classified into different genotypes depending on the combination of alleles in three polymorphic regions: the signal (s), intermediate (i) and mid (m) regions. Different forms vary in activity and toxicity, with s1/i1/m1 types being the most active and toxic to a wide range of cell types, whereas s2/i2/m2 types are less active and virtually non‐toxic (Atherton *et al*., 1995; Letley *et al*., 2003; Rhead *et al*., 2007). The more active and toxic *vacA* genotypes are also more commonly associated with disease (Rhead *et al*., 2007; Basso *et al*., 2008).

The factors determining *vacA* gene expression are not fully understood, and the mechanisms of its regulation are elusive. We have identified a potential stem‐loop forming structure in the 5′ untranslated region (UTR) of the *vacA* gene. As such structures have been found to be associated with transcript stability in several other bacteria (Emory *et al*., 1992; Bricker and Belasco, 1999; Unniraman *et al*., 2002), we sought to explore the influence of this structure on *vacA* expression, using a site‐directed mutagenesis approach. We also wanted to explore the effects of environmental stress on *vacA* expression and examine the potential influence of the stem‐loop structure under such stressed conditions. *H. pylori* is likely to encounter a variety of environmental conditions in the gastric niche, and changes in gene expression are necessary for adaption to such conditions. Other groups have also investigated *vacA* expression, and while features of the *vacA* promoter and the transcriptional start point (TSP) of *vacA* have been clearly defined (Forsyth and Cover, 1999), the regulation of *vacA* in response to environmental changes and the underlying reasons for naturally occurring variation in *vacA* expression between strains are poorly understood.

Here we show that a well‐conserved 5′ stem‐loop structure in the UTR of *vacA* mRNA increases the amount of *vacA* mRNA through conferring stability to the transcript leading to more VacA protein and so more toxicity. We also show that under two different types of environmental stress the presence of the stem‐loop is important for maintaining *vacA* mRNA levels.

## Results

### The *vacA* 5′ UTR contains a potential stem‐loop structure that is highly conserved in clinical isolates of *H* *. pylori*

The nucleotide sequences of the *vacA* promoter and 5′ UTR of 67 *H. pylori* strains isolated from gastric biopsies of infected patients were aligned (Figure S1) and the conserved promoter elements −35 region, −10 region and the TSP were identified and confirmed according to previous reports (Forsyth and Cover, 1999). A schematic representation of the *vacA* promoter region, 5′ UTR and open reading frame (ORF) is shown in Figure S2. Upon sequence inspection, we identified a well‐conserved, potential mRNA stem‐loop forming structure between position +4 and +30 in the UTR (Figure S3), confirming previous findings from our group (Masters *et al*., 2008). In strain 60190, the last nucleotide of the stem‐loop sequence is located 80 nucleotides upstream of the first nucleotide of the ribosomal binding site and 89 nucleotides upstream of the *vacA* start codon.

**Figure 1:**
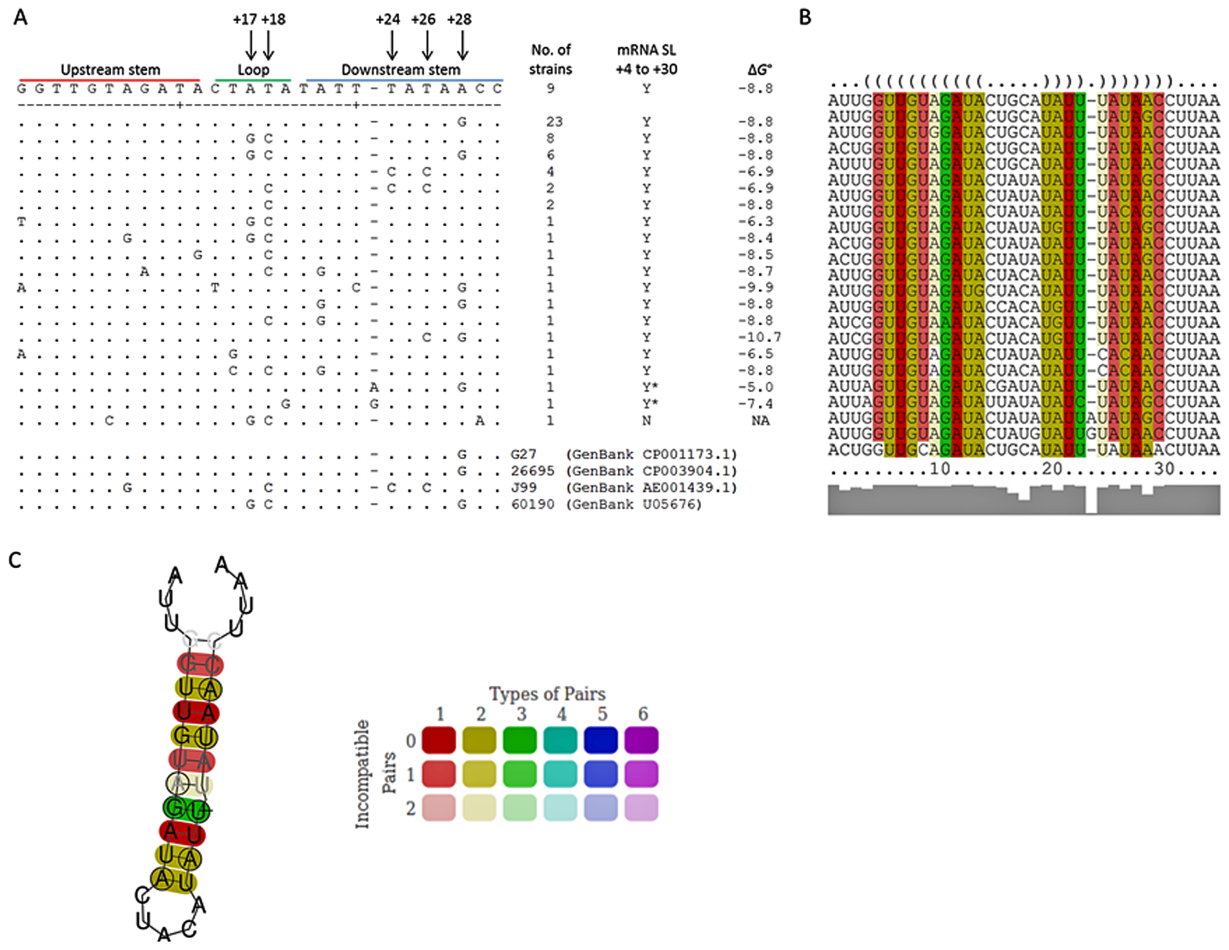
Nucleotide sequences and prevalence of 20 different stem‐loop variants in 67 clinical *H* *. pylori* isolates. A. The +4 to +30 nucleotide stem‐loop DNA sequences from 67 clinical *H* *. pylori* isolates were aligned. The upstream‐, loop‐ and downstream stem are marked by red, green and blue lines respectively. Positions of common polymorphisms are indicated by numbered arrows. The prevalence of each variant is indicated by the number of strains in the right hand column. The ability of the mRNA to form a stem‐loop structure at the +4 to +30 position is indicated by Y (yes) and N (no). The asterisk denotes minor variation in the stem of the +4 to +30 structure. The strain lacking a stem‐loop at position +4 to +30 was predicted to form a small alternative stem‐loop at position +7 to +19. The predicted minimum free energy (Δ*G*°) values for each structure, determined using the online Fold RNAstructure webserver (Reuter and Mathews, 2010) for each variant are also indicated (NA = not applicable). For comparison, the equivalent stem‐loop sequences in four commonly used reference strains of *H* *. pylori* are also included. These are represented among the clinical varieties as well. B. Alignment of each stem‐loop variant (position +1 to +34) subjected to consensus secondary structure analysis by the Freiburg Tools online webserver LocARNA (Smith *et al*., 2010). Nucleotide conservation is indicated by colour, see guide in (C). C. Consensus secondary structure of stem‐loop variants, generated by the Freiburg Tools online webserver LocARNA (Smith *et al*., 2010). Circled nucleotides indicate positions were base substitutions have occurred but base pairing has been maintained (consistent mutations). Grey lettering represents sites where base changes have disrupted base pairing and the colour coding reflects the number of base pairing types at that position and the number of incompatible base changes as given in the colour legend.

Comparison of the potential stem‐loop forming sequence between the strains showed that it is relatively conserved. Within the 67 clinical *H. pylori* isolates in this study, there were 20 different stem‐loop variants (Fig. 1A). Using mRNA secondary structure prediction software (Reuter and Mathews, 2010), all of these variants should be capable of forming mRNA stem‐loop structures (Fig. 1A). The sequences of this region from commonly studied strains G27, 26695, J99 and 60190 were of the most frequent types among the clinical isolates, and all are predicted to form a stem‐loop. One strain, clinical isolate 733, was predicted to have a smaller alternative stem‐loop located at a different position (+7 to +19) with a minimum free energy (ΔG^o^) of −4.6 (Figure S4). Analysis using the consensus mRNA secondary structure prediction software LocARNA (Smith *et al*., 2010), also illustrated the high level of conservation of the stem‐loop (Fig. 1B and C). Commonly, where single base substitutions have occurred, base pairing ability has often been maintained, suggesting the importance of the structure. We identified one common two‐base polymorphism in the loop region (positions +17 and +18) and one common adenine (A) /guanine (G) polymorphism at position +28. The two‐base polymorphism in the loop region is unlikely to affect stability of the loop. However, as the G/A polymorphism is located near the base of the potential stem‐loop, we hypothesised that this may be important for mRNA stability. At this locus, A, compared with G, would give rise to a stronger bond when base pairing with uracil (U) on the opposite arm of the stem‐loop, thus anchoring the stem‐loop more effectively. There were other base substitutions as well, but they were uncommon and often unique to individual strains. The G/A polymorphism at position +28 was the most widespread base substitution and therefore potentially more likely to be of clinical importance.

Additional gastric biopsy samples were collected from the gastric mucosa of 28 of the patients whose *H. pylori* isolates were analysed for *vacA* sequence polymorphisms. These samples were assayed for *vacA* mRNA by RT‐qPCR, and as we and others have found (Forsyth *et al*., 1998; Ayala *et al*., 2004), there was a very broad range, even among those predicted to form the same stem‐loop structure. The relative expression levels ranged from 0.43 to 3.85 in the corpus and from 0.2 to 3.99 in the antrum. There was no direct correlation between stem‐loop sequence and mRNA level. Even among the eight strains with the most common stem‐loop sequence, the range of expression levels in the corpus was 0.51–3.86. This variation may be driven by host factors and/or other genetic differences in the strain. Interestingly, tissue from the patient infected with isolate 733 (alternative stem‐loop) contained the lowest *in vivo vacA* mRNA level of all in the corpus (0.43) and the fifth lowest in the antrum (0.60). Like all other groups working on VacA, we are unfortunately unable to measure protein levels *in vivo* due to cross‐reactivity of all available antibodies. We have, however, compared VacA protein expression between the 60190 wild‐type (WT) strain and isolate 733 by western blotting *in vitro* (Figure S5). Upon densitometry analysis, isolate 733 produced a 1.5‐fold lower relative quantity of VacA, but we cannot exclude the possibility that this may also reflect differences in the promoter or coding sequence.

### Generation of *H* *. pylori vacA* 5′ UTR stem‐loop mutants

**Figure 2:**
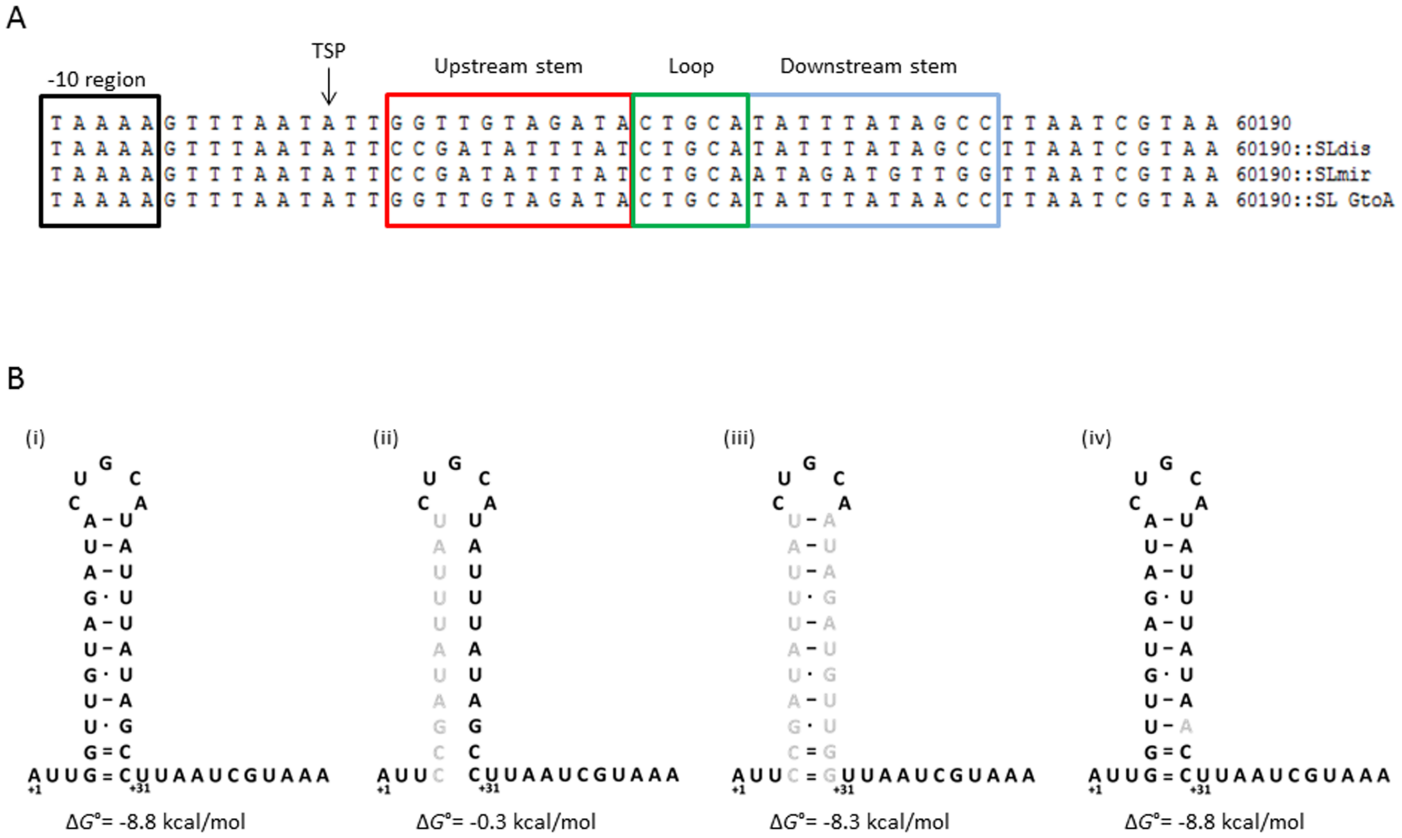
*H* *. pylori* 60190 wild type and stem‐loop mutants. A. DNA sequence alignment of *H* *. pylori* 60190 wild type and mutant strains. The inverted stem‐loop forming structure is marked by coloured boxes: red for the upstream side of the stem, green for the loop and blue for the downstream side. The −10 region (Pribnow box) and the transcriptional start point (TSP) are also indicated. B. Schematic representation of *vac* *A* mRNA 5′ UTR stem‐loop structure in (i) 60190 (wild type), (ii) 60190::SLdis, (iii) 60190::SLmir and (iv) 60190::SLGtoA. Mutational changes are shown in grey. Binding strength between pairing nucleotides is indicated by = (strong), – (relatively strong) and · (relatively weak). The predicted minimum free energy values for each mutant structure, determined using the online Fold RNAstructure webserver (Reuter and Mathews, 2010), are indicated below the schematic representations.

Having observed the wide variation in *vacA* expression between clinical isolates, and to avoid confounding effects from other genetic variation, we went on to examine the influence of the *vacA* 5′ UTR potential stem‐loop structure on *vacA* expression levels in an isogenic background. We used a site directed mutagenesis approach and generated three mutant strains in the chromosomal *vacA* gene in two *H. pylori* strain backgrounds 60190 and SS1 (Fig. 2A). The integration of a chloramphenicol resistance cassette (chloramphenicol acetyltransferase, *cat*) upstream of the *vacA* promoter region was used for antibiotic selection of mutant clones. In order to control for the presence of the *cat* cassette in mutant clones, WT control strains containing the *cat* cassette in the same chromosomal location were also constructed.

For the first mutant (SLdis), we changed the upstream bases of the stem‐loop, preventing base pairing with the downstream bases, thereby disrupting the formation of the potential stem‐loop structure in the mRNA [Fig. 2B(ii)]. To determine whether any potential differences between this mutant and the WT were due to the primary DNA sequence or the secondary mRNA structure, we also generated a mirrored mutant (SLmir) [Fig. 2B(iii)]. In this, the downstream bases were changed to allow base pairing with the changed upstream bases, restoring stem‐loop structure formation but with a different primary nucleotide sequence to WT. In order to determine the importance of the potentially stabilising, naturally occurring +28 base polymorphism within the stem‐loop structure identified from sequencing of clinical *H. pylori* isolates, a mutant (SLGtoA) was constructed where the guanine at position +28 was changed to adenine [Fig. 2B(iv)].

### Disruption of the *vacA* 5′ UTR stem‐loop structure reduces steady‐state mRNA expression levels *in vitro*

**Figure 3:**
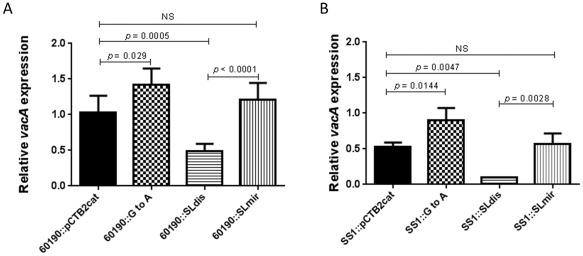
Steady‐state *vac* *A* mRNA levels in *H* *. pylori* stem‐loop mutants. Growth from 24 hour BA‐plates was used to inoculate 10 ml F12 Ham medium (supplemented with 10% FCS and 1% L‐glutamine). Cultures were incubated for 30 min in a microaerobic workstation with shaking at 200 r.p.m., after which samples were collected. RNA was extracted and used as template for cDNA synthesis using reverse transcriptase. RT‐qPCR was then used to determine expression of *vac* *A* mRNA relative to 16s rRNA expression for wild type and mutant strains in (A) *H* *. pylori* 60190 and (B) *H* *. pylori* SS1. A standard preparation of cDNA from the 60190::pCTB2cat strain was used as the comparator in all RT‐qPCR assays. The graphs show the mean (+ SD) *vac* *A* mRNA level of at least four independent experiments. A one‐way ANOVA with Tukey's multiple comparison was used for statistical analysis. NS = not statistically significant.

First, we investigated the influence of the *vacA* 5′ UTR stem‐loop structure on *vacA* mRNA expression levels in our panel of mutants using RT‐qPCR (Fig. 3).

In the SLdis mutant, *vacA* mRNA levels were significantly reduced 2.1‐fold (*P* = 0.0005) and 5.3‐fold (*P* = 0.0047) in strain backgrounds 60190 and SS1 respectively, compared with WT. In the SLmir mutant, where the secondary structure but not the primary sequence matched the WT, *vacA* mRNA expression was similar to that of the WT in both strain backgrounds. *vacA* mRNA expression in the SLGtoA mutant, with potentially increased stem‐loop stability, was increased 1.4‐fold (*P* = 0.029) and 1.2‐fold (*P* = 0.0144) compared with the WT in the 60190 and SS1 strain backgrounds respectively. Due to the higher overall expression levels in strain 60190, we chose to use the mutant strains generated in this background for our subsequent investigations.

### Disruption of the *vacA* 5′ UTR stem‐loop structure reduces VacA protein levels and toxin activity *in vitro*

**Figure 4:**
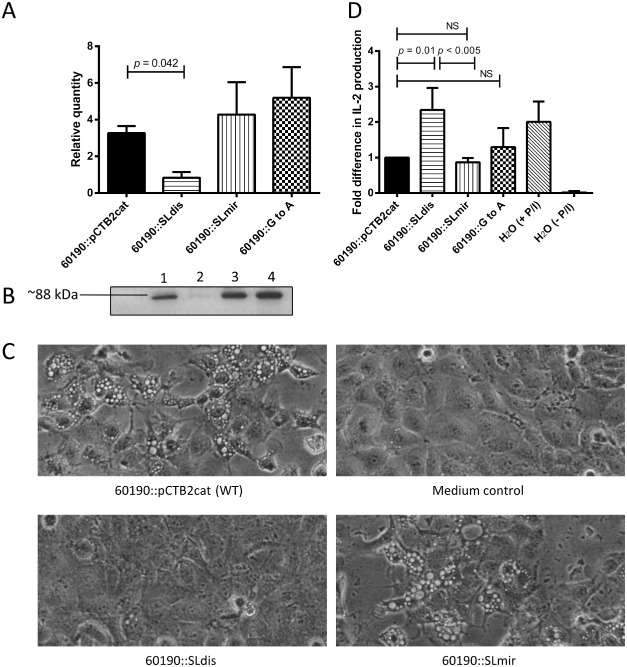
VacA protein levels in *H* *. pylori* 60190 stem‐loop mutants. A. Relative VacA protein quantity in extracts from the 60190 wild type and stem‐loop mutants, determined by densitometry readings from three independent western blots probed with a mixture of rabbit polyclonal anti‐VacA p33 and p55 subunits and horseradish peroxidase‐conjugated goat anti‐rabbit secondary antibody, visualised using chemiluminescence. B. An example western blot, lane 1 = 60190::pCTB2cat, 2 = 60190::SLdis, 3 = 60190::SLmir, 4 = 60190::SLGtoA. C. Vacuolation of RK13 cells incubated with protein extracts of the *H* *. pylori* 60190 *vac* *A* stem‐loop mutants. RK13 cells were incubated with protein extracts (0.2 mg ml^−1^) from wild type (60190::pCTB2cat) and each mutant strain. A negative control containing medium only was also included. After 24 h cells were examined for vacuolation by microscopy (original magnification ×20) and representative photographs taken. D. IL‐2 production in Jurkat T‐cells after incubation with *H* *pylori* 60190 *vacA* stem‐loop mutants. Jurkat T‐cells were incubated with protein extracts (0.2 mg ml^−1^) from wild type (60190::pCTB2cat) and mutant strains for 1 h, after which PMA (P) and ionomycin (I) were added to stimulate cell proliferation. Controls were treated with P/I but no *H* *. pylori* protein extract [H_2_O (+ P/I)] or completely untreated [H_2_O (‐P/I)]. Cells were further incubated for 24 h before 100 μl of each sample was removed and centrifuged. IL‐2 levels in the remaining supernatants were then determined using ELISA. The graph shows the mean (+ SD) IL‐2 levels normalised to wild type (60190::pCTB2cat) of three independent experiments, each performed in triplicate. An ordinary one way ANOVA with Tukey's multiple comparison was used for statistical analysis. NS = not statistically significant.

To determine if the differences observed in mRNA expression levels were also present at the protein level, we prepared protein extracts from WT and mutant strains and assessed differences in VacA protein levels by densitometry (Figs 4A and B and S5). Using SDS‐PAGE and western blotting, 4.0‐fold less VacA protein could be detected in extracts from the SLdis mutant compared with the WT (*P* = 0.042). Consistent with the mRNA findings, there were no significant differences in the VacA protein levels of the SLmir mutant and the WT. The SLGtoA mutant produced a 1.5‐fold higher amount of VacA protein than the WT; however, this difference was not statistically significant (*P* = 0.093).

Next, we investigated whether the differences in protein levels in turn affected total toxin activity. RK13 cells were incubated for 24 h with protein extracts of WT and mutant strains and then examined for vacuolation by microscopy. As expected, the vacuolating activities of extracts were markedly affected by mutations to the stem‐loop (Fig. 4C). RK13 cells incubated with a protein extract of the WT strain were strongly vacuolated after 24 h, compared with untreated control cells. Consistent with the similar level of VacA protein production, cells incubated with protein extracts of the SLmir mutant were also strongly vacuolated. The same was true for the SLGtoA mutant (not shown). Cells incubated with protein extracts from the SLdis mutant, however, did not show any visible signs of vacuolation, consistent with the reduced level of VacA protein.

To further examine the effects of mutating the stem‐loop on VacA protein levels and subsequent cellular activities, we used a Jurkat T‐cell assay (Fig. 4D). As expected, there were no detectable interleukin‐2 (IL‐2) levels in the unstimulated control cells, whereas stimulated control cells produced high levels of IL‐2. IL‐2 levels in cells incubated with WT protein extract were reduced 2.5‐fold compared with the stimulated control cells. In cells incubated with a protein extract of the SLdis mutant, IL‐2 levels were significantly higher (*P* = 0.01) compared with those incubated with WT, indicating a lesser degree of inhibition. When cells were incubated with protein extracts of the SLmir mutant and the SLGtoA mutant, the levels of IL‐2 were not significantly different from that induced by WT.

### The *vacA* 5′ UTR stem‐loop structure is a determinant of mRNA half‐life and transcript stability

Having established that the *vacA* 5′ UTR stem‐loop structure affects *vacA* expression in terms of mRNA, protein and toxin activity, we sought to explore the mechanism behind this. We hypothesised that the stem‐loop structure influences the stability of the *vacA* transcript and therefore investigated its effect on mRNA half‐life. WT and mutant strains were treated and incubated as for the steady‐state mRNA assays but after 30 min of initial incubation, rifampicin was added to inhibit transcription. Samples were then taken at given time points and the relative *vacA* mRNA level at each time point determined using RT‐qPCR, allowing the decay of each transcript to be plotted against time and allowing calculation of mRNA half‐life. Example plots for determining mRNA half‐life are shown in Figure S6, along with a table summarising all half‐life data.

**Figure 5:**
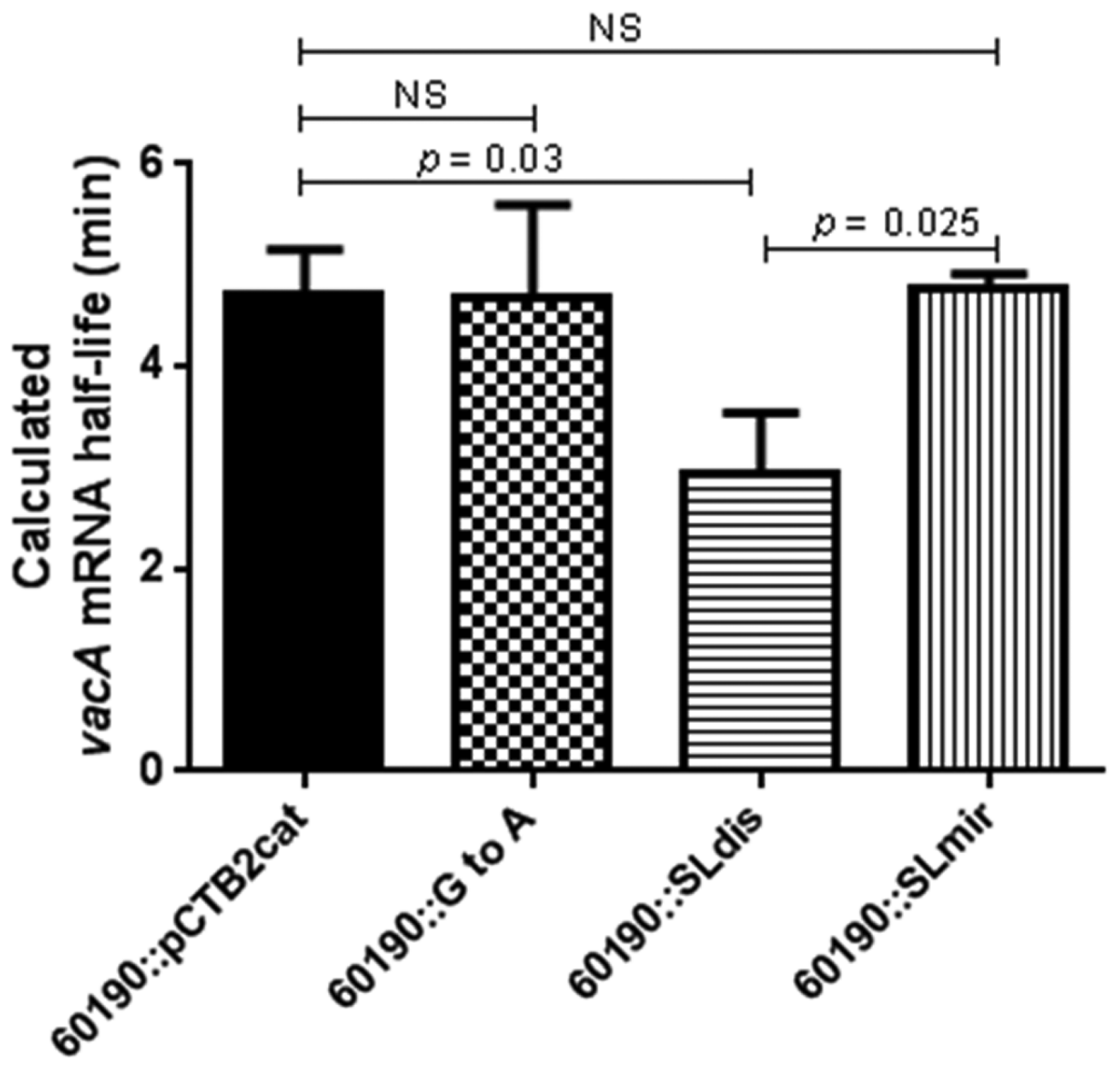
Calculated *vac* *A* mRNA half‐lives of *H* *. pylori* 60190 *vacA* stem‐loop mutants. Growth from 24 h BA‐plates was used to inoculate 10 ml F12 Ham medium (supplemented with 10% FCS and 1% L‐glutamine). Cultures were incubated for 30 min in a microaerobic workstation with shaking at 200 r.p.m., after which rifampicin (100 μg ml^−1^) was added. Samples were then collected at given time points and used for RNA extraction and cDNA synthesis. The *vacA* mRNA expression relative to 16s rRNA expression was determined at each time point and used to plot a decay curve, from which the transcript half‐life for the wild type (60190::pCTB2cat) and each mutant strain could be calculated. The graph shows the mean calculated half‐life (+ SD) of at least three independent experiments. A one‐way ANOVA with Tukey's multiple comparison was used for statistical analysis. NS = not statistically significant.

We found that the *vacA* 5′ UTR stem‐loop structure determined mRNA half‐life and therefore affected the stability of the mRNA (Figs 5 and S6). The mRNA half‐life of the SLdis mutant was significantly reduced 1.6‐fold (*P* = 0.03) compared with WT. The *vacA* mRNA half‐life of the SLmir mutant was similar to that of the WT. This was also the case for the SLGtoA mutant; however, there was a consistent stabilising effect early in the decay curves.

### Environmental stress affects *vacA* expression *in vitro*

**Figure 6:**
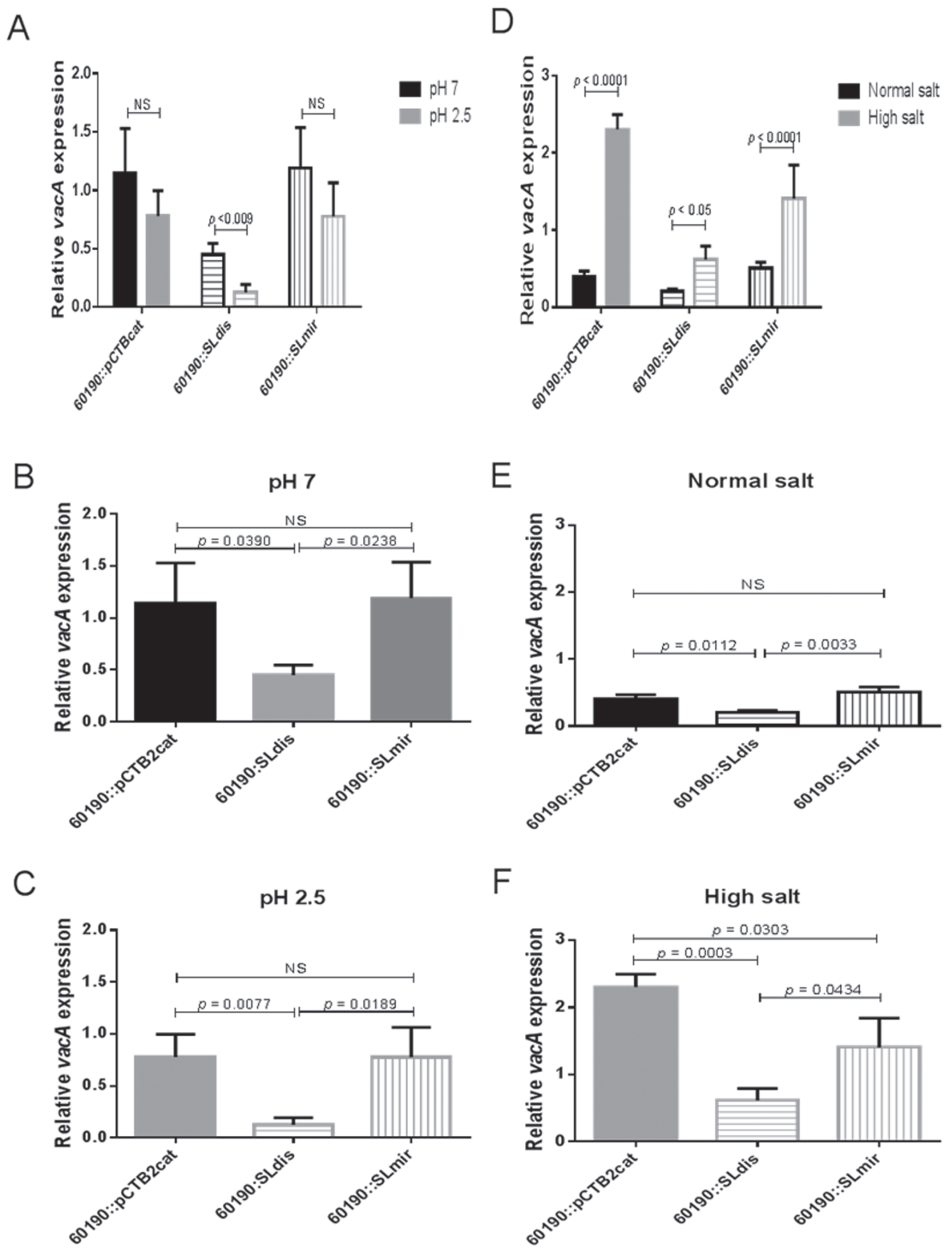
Steady‐state *vac* *A* mRNA levels in *H* *. pylori* 60190 stem‐loop mutants after exposure to environmental stress. A. Steady‐state mRNA levels after exposure to normal and acidic pH. Growth from 24 h BA‐plates was used to inoculate 10 ml normal (pH 7) or acidic (pH 2.5) F12 Ham medium (supplemented with 10% FCS and 1% L‐glutamine). Cultures were incubated for 30 min in a microaerobic workstation with shaking at 200 r.p.m. Samples were collected for RNA extraction and cDNA synthesis performed using reverse transcriptase. RT‐qPCR was then used to determine expression of *vac* *A* mRNA relative to 16s rRNA expression for the wild type (60190::pCTB2cat) and each mutant strain. B. Steady‐state *vacA* mRNA expression levels in *H* *. pylori* 60190 stem‐loop mutants at pH 7. C. Steady‐state *vac* *A* mRNA expression levels in *H* *. pylori* 60190 stem‐loop mutants at pH 2.5. D. Steady‐state *vacA* mRNA levels during normal and high environmental salt conditions. Growth from 24 h BA‐plates was used to inoculate 10 ml F12 Ham medium (supplemented with 10% FCS and 1% L‐glutamine) with normal (8 g l^−1^) or high (33 g l^−1^) NaCl. Cultures were incubated for 60 min in a microaerobic workstation with shaking at 200 r.p.m. Samples were collected for RNA extraction and cDNA synthesis performed using reverse transcriptase. RT‐qPCR was then used to determine expression of *vac* *A* mRNA relative to 16s rRNA expression for the wild type (60190::pCTB2cat) and each mutant strain under the two environmental conditions. E. Steady‐state *vacA* mRNA expression levels in *H* *. pylori* 60190 stem‐loop mutants during normal salt concentration. (F) Steady‐state *vac* *A* mRNA expression levels in *H* *. pylori* 60190 stem‐loop mutants during high salt concentration. All graphs show the mean (+ SD) *vacA* mRNA level of three independent experiments. An unpaired *t*‐test was used for statistical analysis.

In the stomach, *H. pylori* will encounter different environmental conditions and regulate gene expression in response to these in order to adapt. To investigate the effects of environmental stress on *vacA* expression and assess the potential influence of the *vacA* 5′ UTR stem‐loop structure under such conditions, we examined the effects of acidic pH and high environmental salt conditions respectively. We found that both stressful environmental conditions affected *vacA* mRNA expression levels when compared with normal conditions *in vitro* (Fig. 6).

To assess the effect of acid exposure on *vacA* mRNA levels, we included cultures where the growth medium had been adjusted to a pH of 2.5 using concentrated hydrochloric acid. In parallel with cultures incubated in normal (pH 7.0) medium, these were incubated for 30 min before samples were collected for RNA extraction. RT‐qPCR was then used as before to compare *vacA* expression under the different pH conditions. Thirty minutes acid exposure at pH 2.5 reduced *vacA* expression overall compared with that in bacteria grown at pH 7, but expression was only significantly reduced in the SLdis mutant (3.5‐fold reduction, *P* = 0.009) (Fig. 6A). The corresponding fold reductions in the WT strain and mirrored mutant were 1.5 in both strains (not statistically significant). At pH 7, *vacA* mRNA expression in the SLdis mutant was 2.5‐fold less than in WT (*P* = 0.039, Fig. 6B). After exposure to pH 2.5, *vacA* mRNA expression in the SLdis mutant was reduced much more dramatically (6.0‐fold, *P* = 0.0077, Fig. 6C) compared with WT. There was no significant difference in fold expression between the SLmir mutant and the WT at either pH condition (Fig. 6B and C). This experiment shows that the stem‐loop is even more important for maintaining *vacA* mRNA levels after exposure at low pH than it is at normal pH.

For assessing the effect of environmental salt concentrations, the assay was carried out in a similar way as for determining *vacA* mRNA levels under normal conditions, but we also included cultures where the growth medium had been adjusted to a sodium chloride concentration of 33 g l^−1^. Samples for RNA extraction were collected after 60 min of incubation, and RT‐qPCR was used to compare expression levels at normal (8 g l^−1^) and high (33 g l^−1^) salt concentrations. After 60 min of exposure to high salt, *vacA* mRNA levels in WT and mutant strains were significantly increased compared with when incubated for the same time under normal salt conditions (Fig. 6D). The increase in the WT was 5.8‐fold (*P* < 0.0001), whereas in the SLdis mutant, it was 3.1‐fold (*P* < 0.05) and in the SLmir mutant, *vacA* mRNA levels were increased 2.8‐fold (*P* < 0.0001). At normal salt concentrations, *vacA* mRNA levels in the SLdis mutant were reduced 2.0‐fold (*P* = 0.0112, Fig. 6E) in comparison with WT and at high salt concentrations, this difference was again more extreme (3.7‐fold, *P* = 0.0003, Fig. 6F). At normal salt concentration, there was no significant difference between *vacA* mRNA expression levels in the SLmir mutant compared with WT (Fig. 6E); however, there was a statistically significant difference in expression at high salt concentration (*P* = 0.0303, Fig. 6F).

This shows that under environmental conditions leading to high level *vacA* transcription, the stem‐loop is important in maintaining *vacA* mRNA levels.

### In high salt conditions, the *vacA* 5′ UTR stem‐loop structure is important in maintaining *vacA* mRNA stability

**Figure 7:**
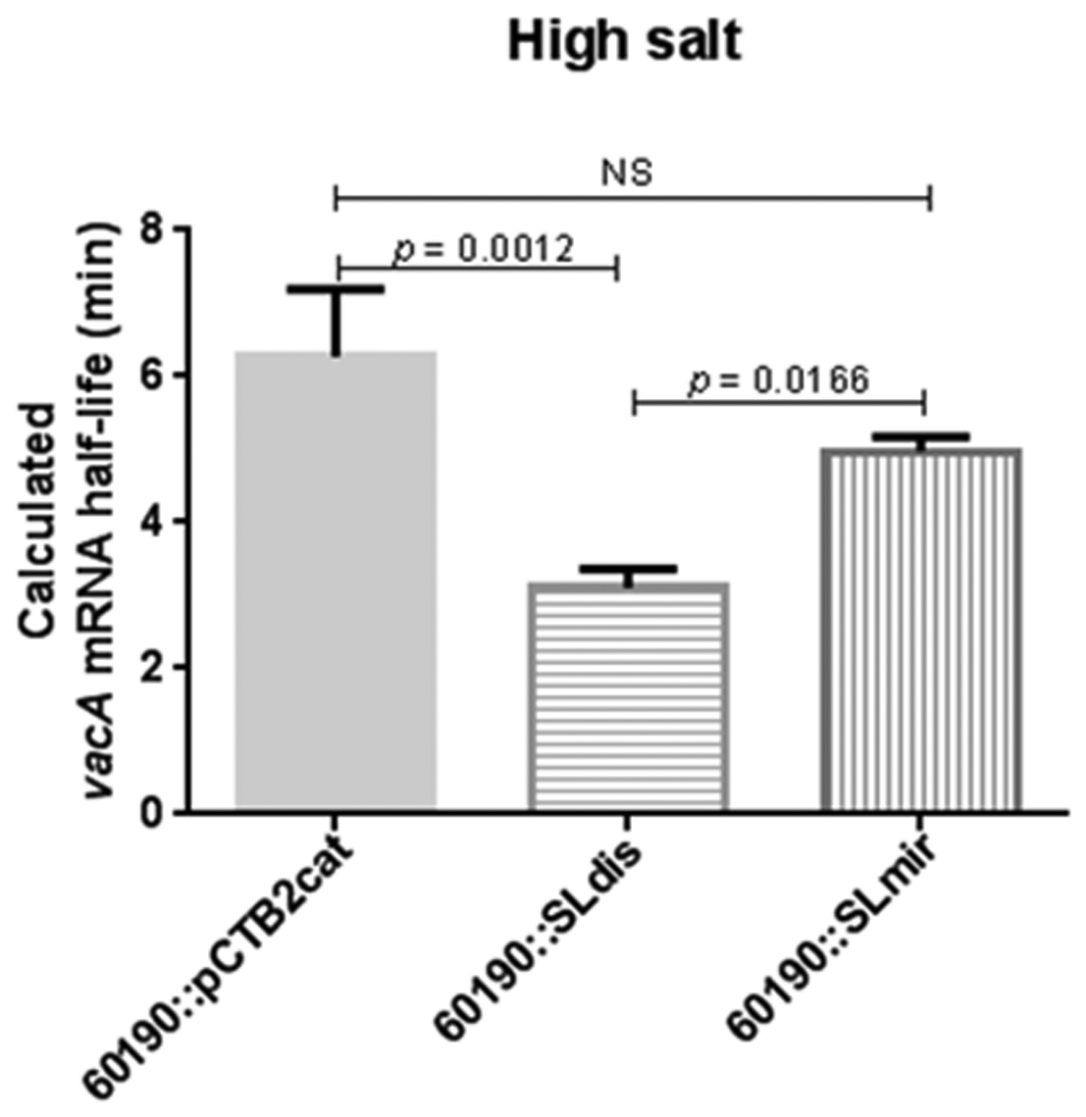
*H* *. pylori* stem‐loop mutants *vacA* mRNA half‐lives during high environmental salt conditions. Growth from 24 h BA‐plates was used to inoculate 10 ml F12 Ham medium (supplemented with 10% FCS and 1% L‐glutamine) with high (33 g l^−1^) NaCl. Cultures were incubated for 60 min in a microaerobic workstation with shaking at 200 r.p.m., after which rifampicin (100 μg ml^−1^) was added. Samples were then collected at given time points and used for RNA extraction and cDNA synthesis. The *vacA* mRNA expression relative to 16s rRNA expression was determined at each time point and used to plot a decay curve, from which the transcript half‐life for the wild type (60190::pCTB2cat) and each mutant strain could be calculated. The graph shows the mean calculated half‐life (+ SD) of at least three independent experiments. A one way ANOVA with Tukey's multiple comparison was used for statistical analysis. NS = not statistically significant.

Having shown that the *vacA* 5′ stem‐loop structure is an important determinant of *vacA* mRNA half‐life, we next aimed to examine the influence of this structure on mRNA stability during environmental stress due to high salt concentrations. The assay was carried out as before, but using F12 Ham medium containing 33 g l^−1^ NaCl. The *vacA* mRNA half‐life for the WT was 6.3 min (Figs 7 and S6). For the SLdis mutant, the *vacA* mRNA half‐life was reduced 2.0‐fold to 3.1 min. Repairing the secondary structure in the SLmir mutant increased the *vacA* mRNA half‐life back to 5.0 min. In comparison with the *vacA* mRNA half‐lives measured for these strains under ‘normal’ salt conditions, the half‐life measured at high salt conditions was significantly higher for the WT (*P* = 0.02), but not for the SLdis mutant showing that the stem‐loop was particularly important in maintaining *vacA* mRNA half‐life under high salt conditions.

## Discussion

Several previous studies have reported the increased stability conferred to mRNA transcripts by the presence of a stem‐loop structure at the 5′ UTR in bacteria (Emory *et al*., 1992; Bricker and Belasco, 1999; Unniraman *et al*., 2002; Mahlen *et al*., 2003). We have identified and characterised such a structure in the *vacA* gene of *H. pylori*. Given that *H. pylori* is the cause of most of the world's peptic ulcer disease and gastric cancer and that VacA is one of its two best recognised virulence factors, this has considerable clinical as well as biological importance.

We identified the stem‐loop through nucleotide sequencing of the *vacA* promoter region of a total of 67 clinical *H. pylori* isolates. In this large group of strains, the stem‐loop sequence was very well conserved (for *H. pylori*) with only 20 different variants. Upon mRNA secondary structure prediction, all variants were likely to form mRNA stem‐loop structures at the same position, apart from in one strain. This is probably because in the few cases where single base substitutions occurred, base pairing was often maintained. In addition, there seemed to be some rescuing mutations in the other arm of the stem. Six strains had two identical base substitutions in comparison with the consensus sequence; thymine (T) (U in the mRNA structure) to cytosine (C) at position +24, and T (U) to C at position +26. The change to a C at position +24, gives rise to mispairing with the adenine at position +8, whereas the change at position +26 gives rise to a stronger C‐G bond when pairing with the guanine at position +10. As these two substitutions are seen in conjunction, it could suggest that the change at position +26 has arisen after the destabilising change at position +24 in order to re‐stabilise the stem‐loop structure. The overall conservation together with the well maintained base pairing ability despite base substitutions and the potential compensatory mutation at position +26 are strong circumstantial evidence as to the importance of this sequence.

We found that *vacA* mRNA levels in the gastric mucosa of infected patients varied widely, which is consistent with previous data (Forsyth *et al*., 1998; Ayala *et al*., 2004). We were unable to explore the role of the stem‐loop in *vacA* expression using clinical isolates as, despite sequence variation, most strains (76%) encoded potential stem‐loop structures with the same predicted stability (ΔG^o^ −8.8). In addition, variables such as other genetic differences between strains, and a differential regulatory response to diverse host gastric environments, confound such an approach. Despite these inherent problems, we did observe that one strain (clinical isolate 733), predicted to be unable to form the stem‐loop at position +4 to +30, but instead form a smaller alternative structure at +7 to +19, had one of the lowest levels of *in vivo vacA* mRNA and somewhat lower VacA protein expression *in vitro*. This encouraged us to explore the role of the stem‐loop structure more thoroughly using an isogenic, site‐directed mutagenesis approach in two different strain backgrounds.

We showed experimentally that disruption of the stem‐loop structure through mutational changes to the upstream bases, preventing base pairing, significantly reduced steady‐state *vacA* mRNA levels, VacA protein levels and mRNA half‐life. We found the same trends in two strain backgrounds; however, the impact of the mutations was more apparent in the SS1 strain than in 60190. We speculate that inter‐strain differences in transcription and RNA degradation machinery may explain this. It is interesting that our disrupted stem‐loop mutant produced much lower VacA protein levels than clinical isolate 733, which is predicted to naturally lack the +4 to +30 stem‐loop structure. We speculate that the predicted small alternative stem‐loop (+7 to +19) in this strain has some mRNA stabilising effect, although we cannot rule out that other genetic differences account for this. Restoration of base pairing through mutational changes to the downstream side of the stem (in the SLmir mutant) resulted in steady‐state mRNA levels, protein levels and mRNA half‐life similar to WT levels, indicating that the stabilising effect of the stem‐loop is due to the secondary structure rather than the primary sequence. A G/A polymorphism at +28 in the stem‐loop sequence is common, and we initially hypothesised that the base pairing might result in a more stable stem‐loop, and consequently a more stable transcript in our SLGtoA mutants compared with the WT. While RNA folding analysis of the stem‐loops did not predict a difference in ΔG^o^ based on base‐stacking energies within the stem helix, the potential clinical importance of this polymorphism warranted investigation. Indeed, we observed a small increase in steady‐state mRNA level and protein expression. We were unable to detect a significant difference in mRNA half‐life to account for this; however, the rifampicin assays may not have been sufficiently sensitive. Although the calculated half‐life was not significantly different for the SLGtoA mutant, we consistently found a slight early effect on stability in the decay curves (Figure S6), which we are now investigating further. Given that *H. pylori* establishes a chronic infection, we speculate that even small differences in toxin production may have important clinical relevance over many decades.

In *E. coli*, stem‐loop structures at the 5′ end of mRNA transcripts increase stability by impeding access of RNA pyrophosphohydrolase (RppH), an enzyme that initiates RNA decay by converting the triphosphorylated 5′ end of the transcript to a monophosphorylated form, making the transcript more vulnerable to degradation by the main ribonuclease RNAse E (Deana *et al*., 2008). An RppH homologue is predicted in *H. pylori* from nucleotide sequence analysis (Baltrus *et al*., 2009), but there is no RNase E homologue (Tomb *et al*., 1997), and mRNA degradation is believed to be mainly attributed to a small RNase J‐containing degradosome (Redko *et al*., 2013). Studies of RNase J in *Bacillus subtilis*, which also lacks RNase E, have shown that this nuclease has greater affinity towards monophosphorylated 5′ ends of mRNA transcripts and is capable of exonucleatic degradation in a 5′ to 3′ direction (Mathy *et al*., 2007). It has also been shown that 5′ stem‐loop structures in *B. subtilis* impede RppH activity similar to in *E. coli* (Richards *et al*., 2011). We therefore hypothesise that despite the lack of RNase E, the stem‐loop structure in the 5′ UTR of *vacA* in *H. pylori* increases transcript stability by limiting RppH access to the transcript, making it a less efficient substrate for RNase J. Future investigations involving RppH and RNase J could be used to confirm the details of this potential pathway of *vacA* mRNA degradation. RNase J is essential in *H. pylori*; however, in future experiments, we plan to generate a conditional deletion mutant, as described by Redko *et al*. (2013), and determine effects on *vacA* mRNA half‐life.

The presence of a stem‐loop structure in the *vacA* 5′ UTR is of particular interest as *H. pylori* has few traditional regulatory networks (Tomb *et al*., 1997), suggesting the importance of post‐transcriptional regulation. This area has elicited interest recently, with the identification of more than 60 small RNAs in *H. pylori* (Sharma *et al*., 2010). Although specific to *vacA*, our results provide an interesting starting point for further investigation into post‐transcriptional regulation and identification of mRNA degradation pathways in *H. pylori*.

In this paper, we have also shown that the presence of the stabilising stem‐loop structure at the 5′ end of the *vacA* transcript is of particular importance during environmental stress. This is important for *H. pylori* given its niche in the harsh environment of the human stomach. *H. pylori* inhabits a near neutral pH environment in the stomach deep in the gastric mucus layer but is exposed to acidic pH for periods when this barrier is disrupted, for example by passage of food through the stomach. We observed a small but non‐significant decrease in WT *vacA* expression after a short‐term acid shock at pH 2.5. Interestingly, we found that disruption of the stem‐loop structure led to a significant reduction in *vacA* mRNA levels after acid exposure. This suggests that the stability conferred to the transcript by the stem‐loop structure is of extra importance during pH challenge, allowing *vacA* mRNA levels to be maintained despite a potential decrease in active transcription. It is known that the VacA protein undergoes structural changes upon exposure to acid that increase its stability at low pH, make it more resistant to pepsin digestion and increase its toxic activity (de Bernard *et al*., 1995). Therefore, *H. pylori* has adopted multiple strategies to maintain toxin function under acid stress. Maintaining *vacA* mRNA levels and protein stability during pH challenge could potentially be important in aiding bacterial survival in this transiently acidic environment. *In vitro* studies have shown that *vacA* may function as a urea permease, facilitating release of urea from host cells (Tombola *et al*., 2001). It has been shown (Wen *et al*., 2003) that intrabacterial cytoplasmic pH during exposure to a range of different environmental pHs was more effectively buffered in the presence of environmental urea. Taken together, this may suggest the importance of consistent *vacA* expression, aided by the presence of the stem‐loop structure, during environmental pH challenge when the need for urea is increased.

The effect of pH on *vacA* expression has been reported previously, largely based on microarray analysis. Two separate studies (Allan *et al*., 2001; Ang *et al*., 2001) reported no difference in *vacA* expression in response to acid exposure. In these two studies, there was no overlap between the up or downregulated genes identified. However, the culturing of bacteria and type of acid exposure was quite different: one study (Allan *et al*., 2001) analysed changes in gene expression after a brief 30 min exposure to pH 4.0 in citrate buffer, whereas the other (Ang *et al*., 2001) analysed differences in gene expression in bacteria grown for 48 h on agar plates containing acidified media with pH ranging from 5.5 to 7.2. Another microarray study (Merrell *et al*., 2003) showed that *vacA* was repressed after 30, 60, 90 and 120 min in acidified (pH 5) brucella broth. Some of the other acid‐responsive genes identified in this study overlapped with the two previously mentioned studies (Allan *et al*., 2001; Ang *et al*., 2001), but as pointed out by the authors, comparison is difficult due to the differences in culture conditions that could have major impact on growth phase and bacterial physiology (Merrell *et al*., 2003). Interestingly, repression of *vacA* expression at pH 5 has since been confirmed (Bury‐Mone *et al*., 2004). In this study, gene expression was compared between exponentially growing bacteria (one doubling time) at pH 7 or pH 5 using microarray. The results for a selection of genes were then validated using a variety of approaches and for *vacA* this was in line with a previously performed proteome analysis that showed that VacA production was increased at pH7 compared with pH 5 (Jungblut *et al*., 2000).

It is difficult to compare the findings of the above studies with our findings. First of all we have not used microarray based techniques to study the general changes in *H. pylori* gene expression in response to acid. We have investigated the effect of a short‐term, highly acidic exposure on *vacA* gene expression specifically, and characterised it using a highly sensitive real time RT‐qPCR system. Compared with previous studies, we used a much stronger acidic exposure of pH 2.5 to properly stress the bacteria during a short 30 min incubation and to mimic more closely the situation in the human stomach. Our main interest was to simulate an environmental stress that would affect *vacA* expression specifically, and in this sense we were successful.

In addition to pH, we also investigated the effect of salt on *vacA* expression and observed a significant increase in WT *vacA* expression levels after exposure to high environmental salt concentration. These findings are consistent with a previous study that showed that *vacA* expression could be increased in response to NaCl shock depending on the specific strain background (Gancz *et al*., 2008). In our study, the influence of the stem‐loop structure during high levels of *vacA* transcription was less clear‐cut than during low levels of transcription, but it still appeared important. In the WT, *vacA* mRNA levels were increased approximately twice as much as for the disrupted mutant, suggesting that the stem‐loop structure contributes to the high levels of *vacA* mRNA during high salt shock. However, for the mirrored mutant, the fold increase in *vacA* mRNA level was similar to that of the disrupted mutant. This could suggest that during high salt conditions, the primary sequence of the stem‐loop as well as the secondary structure is important for increased *vacA* transcription. We also investigated the effects of high environmental salt conditions on *vacA* mRNA half‐life. This showed that during high salt conditions, *vacA* mRNA half‐life is increased in the WT strain, but not in the disrupted mutant. This suggests that the mechanism behind the increase in *vacA* expression during high salt conditions is increased mRNA stability. The increase in *vacA* expression during high environmental salt conditions is interesting and potentially of pathogenic relevance. There is epidemiological evidence in humans showing that a high salt diet in conjunction with *H. pylori* infection increases the risk of gastric cancer (Tsugane and Sasazuki, 2007; Wang *et al*., 2009; D'Elia *et al*., 2012). Animal studies using Mongolian gerbils have also shown that *H. pylori* infected animals fed a high salt diet are more likely to develop gastric cancer than those fed a regular diet (Gaddy *et al*., 2013). Interestingly, in animals fed the high salt diet, *in vivo cagA* (another major virulence gene of *H. pylori*) transcription levels were higher than in those on a regular diet. This is consistent with previous findings regarding *cagA in vitro* transcription, which is also increased in response to high salt concentrations (Loh *et al*., 2007). Thus, salt appears to increase transcription of two of the major virulence factors of *H. pylori*, and for *vacA* the 5′ stem‐loop in the mRNA appears to contribute to the high mRNA levels.

In summary, we have identified a stabilising stem‐loop structure in the *vacA* 5′ UTR that contributes to maintaining *vacA* mRNA levels during conditions of environmental stress. *In vivo*, we confirmed the finding of others that *vacA* mRNA levels vary widely and this variation was not easily explained by differences in predicted *vacA* mRNA stability. In contrast, we found the stem‐loop structure to be extremely well‐conserved and present in virtually all strains. When the stem‐loop was disrupted in two individual strain backgrounds, *vacA* mRNA and protein expression was reduced, as well as mRNA half‐life. We therefore propose that presence of the stem‐loop structure is beneficial and usually assists in maintaining expression of the toxin via enhancement of transcript stability, especially during adverse environmental conditions. This is important for colonisation, as we and others have previously shown that *vacA* null mutants are less able to colonise mice (Salama *et al*., 2001; Winter *et al*., 2014b). In isogenic mutants, the common G to A polymorphism resulted in slightly increased expression of the toxin. There was also a consistent early stabilising effect on the *vacA* transcript, which requires further investigation. In future studies, we also aim to determine whether the effects arising from stem‐loop sequence polymorphisms have an impact in an animal model. We recently reported that colonisation of mice with mutants expressing more active forms of VacA increased the development of pre‐cancerous metaplasia in the stomach (Winter *et al*., 2014b). It would therefore be interesting to investigate the effect of high *vacA* transcription levels on the development of this metaplasia, potentially through upregulation of transcription using a high‐salt diet.

## Experimental procedures

### Bacterial strains and growth conditions

*Helicobacter pylori* strains used for mutagenesis studies were 60190 (*cag* PAI^+^, *vacA* ^+^ s1/i1/m1) and SS1 (*cag* PAI^−,^ *vacA* ^+^ s2/i2/m2). Mutants constructed are listed in Table 1. Bacterial strains were maintained on commercially prepared blood agar base#2 (BA) plates containing 5% horse blood (Oxoid, UK) and grown in a MACS VA500 microaerobic workstation (Don Whitley Scientific, UK) at 37°C in a humidified atmosphere containing the following gas composition: 86% nitrogen, 6% oxygen, 3% hydrogen and 5% carbon dioxide. Where appropriate, chloramphenicol (Sigma‐Aldrich, UK) (30 μg ml^−1^) was added to in‐house prepared blood agar base#2 plates supplemented with 5% defibrinated horse blood (Oxoid, UK). All strains were passaged minimally.

**Table 1:** Mutant strains used in this study, based on *H* *. pylori* 60190 and SS1 parental strains

Mutant strain	Characteristics	Source
pCTB2cat (WT)	As 60190/SS1 except chloramphenicol acetyltransferase gene (*cat*) inserted immediately 3′ to *cys*S; Cm^R^	This study
SLdis	As 60190/SS1::pCTB2cat but 11 nt exchanged between position +4 to +14 (included) in the *vacA* 5′ UTR.	This study
SLmir	As 60190/SS1::SLdis but 11 nt exchanged between position +20 and +30 (included) in the *vacA* 5′ UTR.	This study
SLGtoA	As 60190/SS1::pCTB2cat but A instead of G at position +28 in the *vacA* 5′ UTR.	This study

### Site‐directed mutagenesis

Plasmid constructs carrying the desired mutational changes were generated in the plasmid pCTB2cat (Letley *et al*., 2003) using the QuikChange II XL Site‐Directed Mutagenesis kit (Agilent Technologies, UK) according to the manufacturer's protocol with the primers listed in Table 2. pCTB2cat contains the first 274 bp of *vacA* from strain 60190 together with the last 0.6 kb of the upstream gene *cysS*, the 229 bp intergenic region and a chloramphenicol resistance cassette (chloramphenicol acetyltransferase, *cat*) inserted immediately 3′ to *cysS*, upstream of the *vacA* promoter. Mutant plasmid constructs were introduced into *H. pylori* strain 60190 and *H. pylori* strain SS1 using natural transformation and electroporation respectively. The *vacA* 5′ UTR stem‐loop sequence of strain SS1 is the same as the most common type shown in Fig. 1 and differs to that of strain 60190 by just two nucleotide substitutions within the loop region. The SS1 stem‐loop mutations were based on the 60190 *vacA* 5′ UTR sequence, with the aim of confirming the effect of the same stem‐loop variants on *vacA* expression in a different genetic background. Mutant strains that had acquired the chloramphenicol resistance cassette were rescued on chloramphenicol plates. Under these conditions, *H. pylori* invariably integrates the cassette into its chromosome by double cross‐over homologous recombination. For each mutation, introduction into the chromosomal *vacA* gene was confirmed by nucleotide sequence analysis, which also meant that the precise cross‐over points could be checked. WT control strains containing the *cat* cassette at the same location upstream of the *vacA* promoter were also constructed by transforming 60190 and SS1 with pCTB2cat to control for any effect of the *cat* cassette on *vacA* expression.

**Table 2:** Primer sequences

Primer name	Primer sequence (5′‐3′)	Source
A3436 (VA1‐F)	ATGGAAATACAACAAACACAC	Atherton *et al*. (1995)
C1226 (VA1‐R)	CTGCTTGAATGCGCCAAAC	Atherton *et al*. (1995)
DL1	GCTTTGATGGACACCCCACAAGG	Aviles‐Jimenez *et al*. (2004)
VacR10	TTATAAGTCCCTACAGCGTC	This study
16s rRNA F	CGATGAAGCTTCTAGCTTGC	Narayanan (2005)
16s rRNA R	ATAGGACATAGGCTGATCTC	Narayanan (2005)
SLdisF	TGATAAAAGTTTAATATTCCGATATTTATCTGCATATTTATAGCC	This study
SLdisR	GGCTATAAATATGCAGATAAATATCGGAATATTAAACTTTTATCA	This study
SLmirF	TCCGATATTTATCTGCAATAGATGTTGGTTAATCGTAAATGCAACAG	This study
SLmirR	CTGTTGCATTTACGATTAACCAACATCTATTGCAGATAAATATCGGA	This study

Underlined nucleotides represent mutational changes in *H. pylori* strain 60190.

### Collection of patient samples and isolation of clinical *H* *. pylori* strains

Gastric antrum and corpus biopsies were collected from patients undergoing a routine upper GI endoscopy at the Queen's Medical Centre, Nottingham, UK, with written informed patient consent and approval from Nottingham Research Ethics Committee 2. For growth and isolation of infecting *H. pylori* strains, biopsies were placed into isosensitest broth (Oxoid, UK) containing 15% (v/v) glycerol (Sigma‐Aldrich) immediately after collection and then used to inoculate BA plates. For mRNA analysis, biopsies were treated with RNALater (Sigma‐Aldrich) and stored at −80^o^C prior to use.

### Steady‐state mRNA assays

Strains were grown on BA plates as described above for 24 h. Growth from the outer edges of two plates was harvested into 5 ml of F12 Ham medium (supplemented with 10% foetal calf serum (FCS) and 2 mM L‐glutamine; all Sigma‐Aldrich).This suspension was used to inoculate 10 ml of the same medium at a starting optical density (λ = 600 nm) (OD_600_) of 0.1. Cultures were incubated for 30 min in a microaerobic workstation with shaking at 200 r.p.m., after which 1 ml samples were collected and transferred to 2 volumes of RNAprotect Bacteria Reagent (Qiagen, UK). Samples were vortexed for 5 s and incubated at room temperature for 5 min before centrifugation at 5000 *g* for 10 min, according to the manufacturer's recommendation. The remaining cell pellets were stored at −80°C and used for RNA extraction.

### mRNA decay assays and calculation of mRNA half‐lives

For RNA decay assays, strains were grown, harvested and used to inoculate 10 ml of supplemented F12 Ham medium as described for steady‐state mRNA assay above. After 30 min of initial incubation, rifampicin (Sigma‐Aldrich) (100 μg ml^−1^) was added to the culture in order to inhibit transcription. Cultures were further incubated, and 1 ml samples were collected at 1.5, 2.5, 5 and 10 min post rifampicin addition. Samples were transferred to RNAprotect Bacteria Reagent (Qiagen) and processed and stored as described above according to the manufacturer's recommendation. RNA extraction, cDNA synthesis and RT‐qPCR (described below) were used to determine the relative *vacA* expression for each strain at the given time points after rifampicin addition. These values were then converted into the natural logarithm (*ln)* and plotted against time. From this plot, the half‐life coefficient *k* was determined and used in the formula T_1/2_ = *ln*(2)/*k* to calculate the *vacA* mRNA half‐life for each strain.

### Exposure of *H* *. pylori* to low pH and high salt concentrations

Strains were grown and harvested into 10 ml aliquots of supplemented F12 Ham medium, at the normal pH (pH 7.0–7.4) and acidified to pH 2.5 with addition of concentrated HCl, at a starting OD_600_ of 0.1. Other paired cultures were similarly prepared using supplemented F12 Ham medium containing 8 g l^−1^ or 33 g l^−1^ sodium chloride (Fisher Chemicals, UK). Cultures were incubated in a microaerobic workstation for 30 min (pH experiments) or 60 min (salt experiments) after which 1 ml samples from each condition were sampled and transferred to RNAprotect Bacteria Reagent (Qiagen). Samples were processed as described previously, according to manufacturer's recommendation and used for RNA extraction.

### RNA extraction and cDNA synthesis

Total RNA from bacterial cultures and human gastric biopsies was extracted using the RNeasy Mini kit (Qiagen). To ensure removal of any residual genomic DNA, extracted RNA was DNase treated using the Ambion DNA‐free kit (Applied Biosystems, USA) before reverse transcription into cDNA using Superscript II and random hexamer primers (both Invitrogen, USA). For each sample, a negative control cDNA reaction containing no reverse transcriptase was performed. This was included in each real‐time RT‐qPCR run to confirm the absence of contaminating genomic DNA.

### RT‐qPCR

Real‐time PCR was performed on triplicate samples using the Rotor‐gene 3000 system (Corbett Research, Australia) and Power SYBR Green Master mix (Applied Biosciences, UK). The primer pairs A3436/C1226 and 16s rRNA F/16s rRNA R were used to amplify a 259 bp *vacA* product and a 164 bp 16s rRNA product respectively (see Table 2 for primer details). The PCR cycling conditions were: 95°C for 10 min followed by 45 cycles of 95°C for 15 s, 56°C for 30 s, 72°C for 30 s. Melt curve analysis was performed to confirm primer specificity, and no template control samples were also included in each run. Amplification efficiencies were calculated using serially diluted genomic 60190 DNA and were 1.82 (*R* ^2^ = 0.99) for *vacA* and 1.63 (*R* ^2^ = 0.99) for 16s rRNA. The Pfaffl method (Pfaffl, 2001) was used to calculate the *vacA* mRNA level in each sample relative to a comparator sample that was included in every run (assigned an arbitrary expression level of 1.0), normalising to expression of the reference gene 16s rRNA. For all samples, the standardised comparator was cDNA synthesised from a single batch of 60190::pCTB2cat (WT) RNA.

### Preparation of protein extracts, SDS‐PAGE and western blotting

Preparation of protein, SDS‐PAGE and western blotting were carried out using a modified protocol of methods described elsewhere (Winter *et al*., 2014b). Strains were grown on BA plates as described above for 24 h, after which growth was harvested into 1 ml of PBS. Cells were pelleted by centrifugation and re‐suspended into 500 μl sterile distilled water then vigorously vortexed and incubated at room temperature for 30 min. Suspensions were then further centrifuged at 10 000 *g* for 5 min and the resulting protein containing supernatants used directly or stored at −20°C. Total protein concentration was determined using a bicinchoninic acid (BCA) assay (Thermo Scientific, USA), and samples were adjusted to concentrations of 1 mg ml^−1^. Samples were boiled in reducing sample buffer containing SDS and dithiothreitol (DTT) before 10 μg of each sample per lane was separated using SDS‐PAGE (12% acrylamide). After transfer to a nitrocellulose membrane, the membrane was stained with Ponceau S solution (Sigma‐Aldrich) to confirm equal loading of gel lanes and protein profiles of the isogenic mutant strains. Western blotting was used to determine the relative VacA protein content using a 1:1 mixture of rabbit polyclonal anti‐VacA p33 and p55 subunits diluted 1:10 000 (generated at the University of Nottingham Biomedical Services Unit) and horseradish peroxidase‐conjugated goat anti‐rabbit secondary antibody (Sigma‐Aldrich), also diluted 1:10 000. Protein bands were visualised using Amersham ECL Prime Western blotting reagent (GE Healthcare). Films were scanned using a GS‐800 Calibrated Densitometer (Bio‐Rad) and data analysed using Quantity One software version 4.6.5 (Bio‐Rad). Densitometry readings were calculated relative to the most prominent non‐specific 58 kDa band, which was common to all lanes and present at similar densities.

### Vacuolation assay

Vacuolation assays were carried out according to an established method (Winter *et al*., 2014a). RK13 cells (American Type Culture Collection CCL‐37) were maintained in F12 Ham medium supplemented with 10% heat inactivated FCS and 1 % L‐glutamine, at 37°C with 5% carbon dioxide. Cells were seeded into a 96 well plate at 0.8 × 10^4^ cells per well and incubated with bacterial protein extracts to a final concentration of 0.2 mg ml^−1^ in the presence of 10 mM ammonium chloride (Sigma‐Aldrich) for 24 h, after which cells were examined for vacuolation under the microscope (×20 objective magnification) and representative photographs taken.

### Jurkat T‐cell proliferation assay and interleukin‐2 ELISA

Assessment of Jurkat T‐cell proliferation and IL‐2 ELISA were carried out according to a previously described method (Winter *et al*., 2014a). Jurkat T‐cells (donated by R. McIntosh, Academic Clinical Oncology Department, University of Nottingham) were maintained in RPMI 1640 medium supplemented with 10% heat inactivated FCS with 5% carbon dioxide at 37°C. Cells were seeded into a 96 well plate at 1 × 10^5^ cells per well and incubated with bacterial protein extracts (0.2 mg ml^−1^) for 1 h, after which PMA (50 ng ml^−1^) and Ionomycin (1 μM) (Sigma‐Aldrich) were added to stimulate cell proliferation. Cells were further incubated for 24 h before 100 μl of each sample was removed and centrifuged. IL‐2 levels in the remaining supernatants were then determined using ELISA (Thermo Scientific).

### Nucleotide sequence analysis

Genomic DNA was extracted from *H. pylori* cultures using a GenElute bacterial genomic DNA kit (Sigma‐Aldrich). PCR using primers DL1 and VacR10 (Table 2) was used to amplify the region between *cysS* and *vacA*, covering the last 25 bp of *cysS*, the 229 bp intergenic region and the first 375 bp of the *vacA* ORF. Amplified products were sequenced commercially by Source Bioscience Life Sciences, Nottingham, UK. MegAlign (DNAStar, Madison, WI) was used to align and analyse the sequence data. Prediction of RNA structures was performed using online RNA Fold or MaxExpect RNAstructure Web server (http://rna.urmc.rochester.edu/RNAstructureWeb) (Reuter and Mathews, 2010). Consensus structures of mRNA were analysed using the online Freiburg Tools LocaRNA webserver (http://rna.informatik.uni‐freiburg.de) (Smith *et al*., 2010).

### Statistical analysis

GraphPad Prism software (version 6) (GraphPad Software, Inc., La Jolla, CA, USA) was used for all statistical analysis. The statistical tests used are indicated in the figure legends. *P* values of ≤ 0.05 were taken to indicate statistically significant differences.

## Supporting information

Supporting Information
