# Supplementary material for: Expression of the Helicobacter pylori virulence factor vacuolating cytotoxin A (vac A) is influenced by a potential stem‐loop structure in the 5′ untranslated region of the transcript
Source: Mol Microbiol. 2015 Sep 10;98(5):831–46. doi: 10.1111/mmi.13160 (PMC4843948; doi:10.1111/mmi.13160)
Supplement: Supplementary file 1 — Supporting Information [file MMI-98-831-s001.pdf]

Supplementary Information

Expression of the *H. pylori* virulence factor vacuolating cytotoxin A (*vacA*) is influenced by a potential stem-loop structure in the 5' untranslated region of the transcript

Karin R. Amilon*, Darren P. Letley, Jody A. Winter**, Karen Robinson and John C. Atherton

Nottingham Digestive Diseases Biomedical Research Unit, School of Medicine, University of Nottingham, Nottingham, UK.

Current addresses: *The Roslin Institute and Royal (Dick) School of Veterinary Studies, University of Edinburgh, Edinburgh, UK. **Pathogen Research Group, School of Science and Technology (Biosciences), Nottingham Trent University, Nottingham, UK.

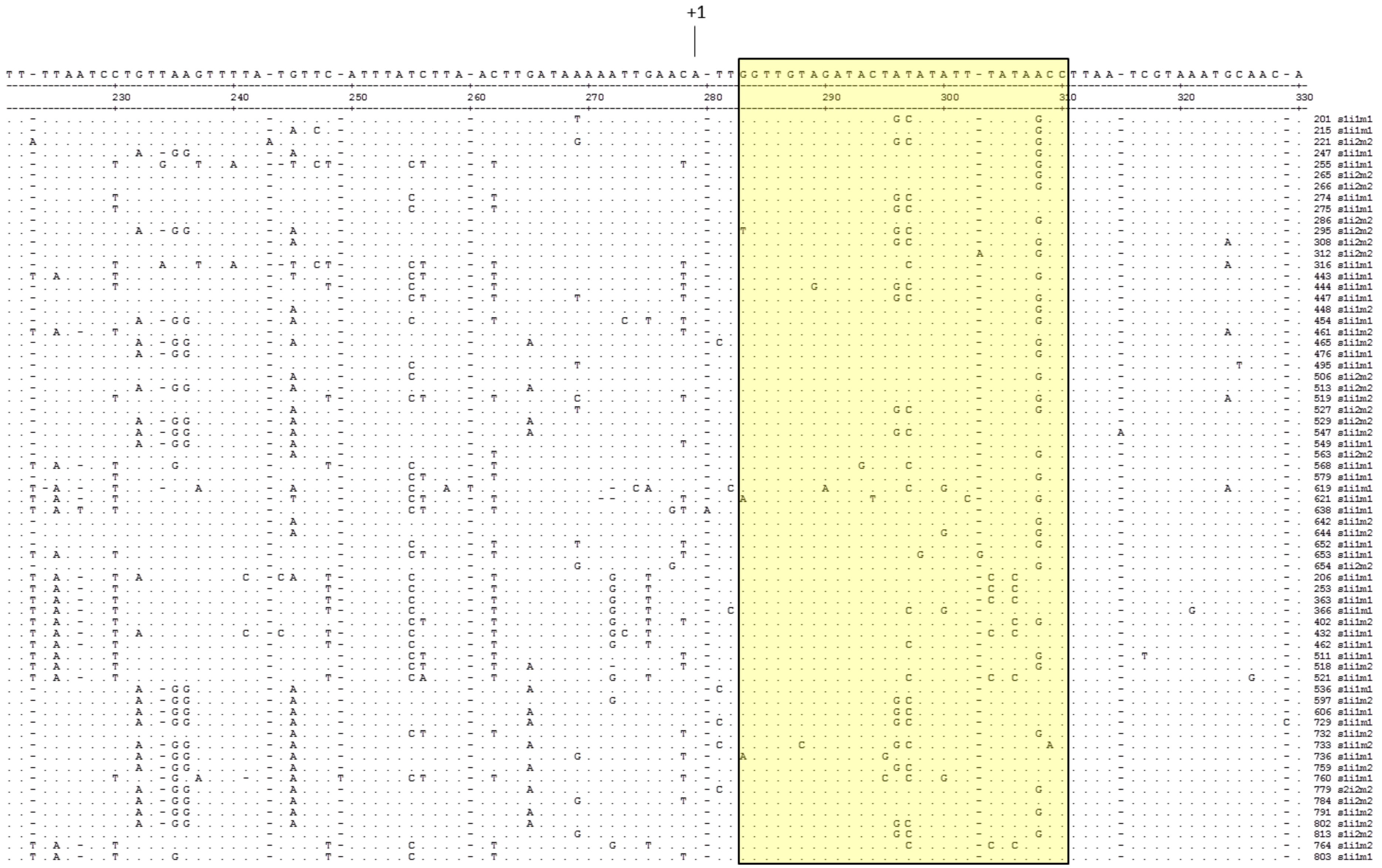

Figure S1. Partial nucleotide sequence alignment of the *vacA* promoter and untranslated region. Genomic DNA extracted from 67 clinical *H. pylori* isolates were subjected to PCR amplification with primers DL1 and VacR10, covering the *vacA* promoter region. Amplified products were sequenced commercially (Source Bioscience Lifesciences Ltd, Nottingham, UK). Sequence data was aligned and analysed using MegAlign (DNAstar Inc.). The conserved sequence of the potential mRNA stem-loop forming structure is indicated by a yellow box. The transcriptional start point (TSP) is marked by a vertical line.

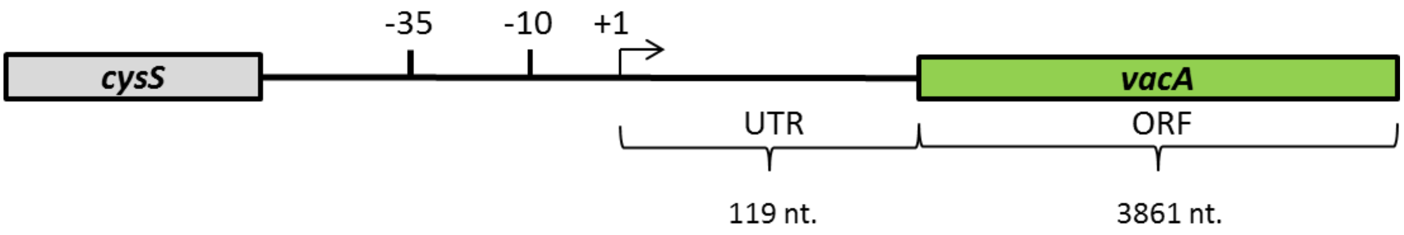

Figure S2. Schematic representation of the *vacA* promoter region and 5’ untranslated region (UTR). The promoter region is located in the intergenic space downstream of the housekeeping gene *cysS* and upstream of the *vacA* open reading frame (ORF). Conserved binding sites for RNA polymerase, the -35 region and -10 region (Pribnow box) are indicated. Nucleotide positions are relative to the transcriptional start point (TSP), which has been assigned +1. The length of the *vacA* 5’ UTR and ORF are indicated by the number of nucleotides (nt).

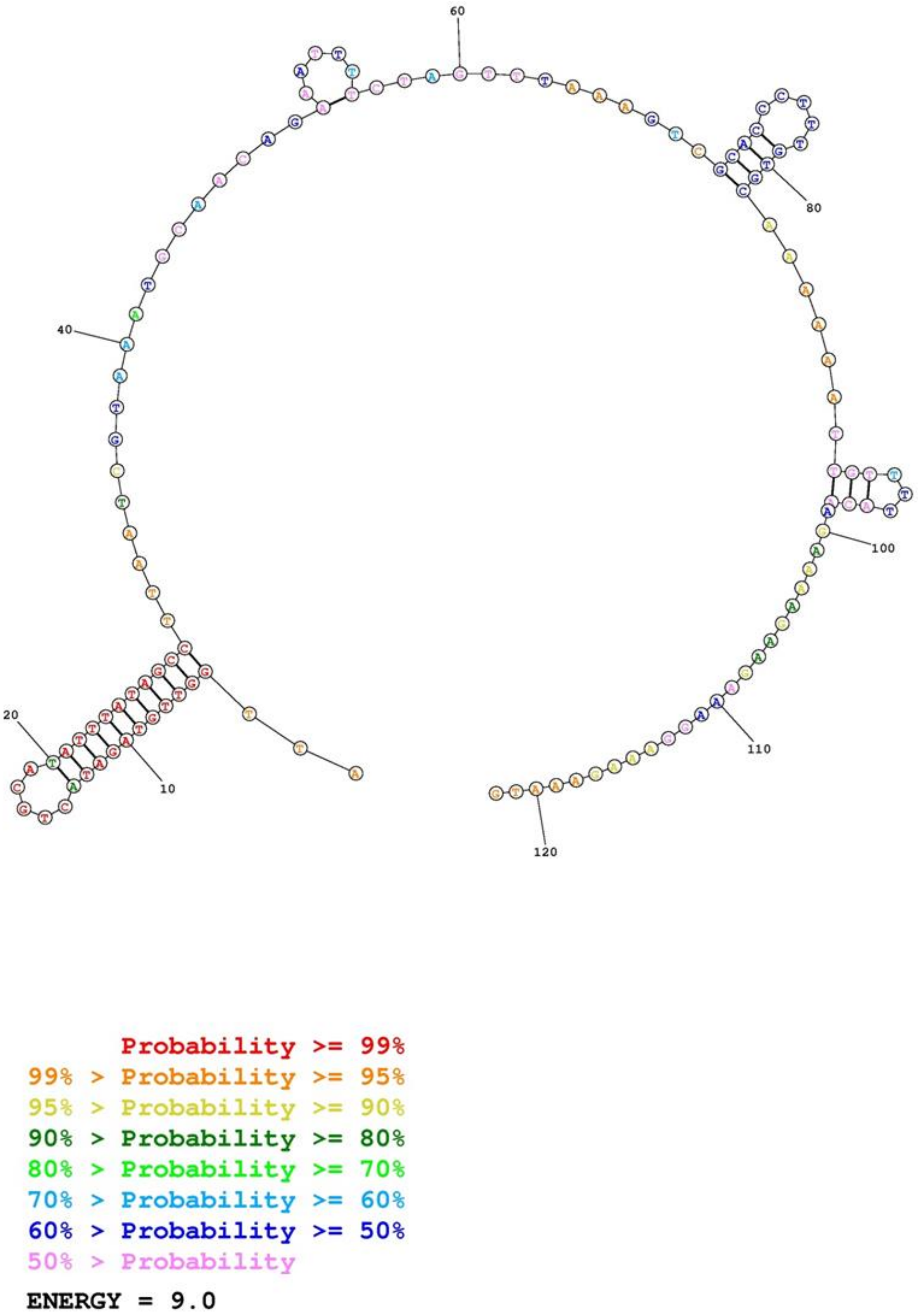

Figure S3. mRNA structure prediction of the *vacA* 5′ UTR in strain 60190.
The nucleotide sequence of the *vacA* 5′ UTR, from the transcriptional start point (TSP) at +1 to the ATG translational start codon was subjected to mRNA structure folding prediction using the online MaxExpect RNAstructure Web server (Reuter & Mathews, 2010). Note the high probability of the stem-loop structure forming and the low probability of formation of any other secondary structures in this region.

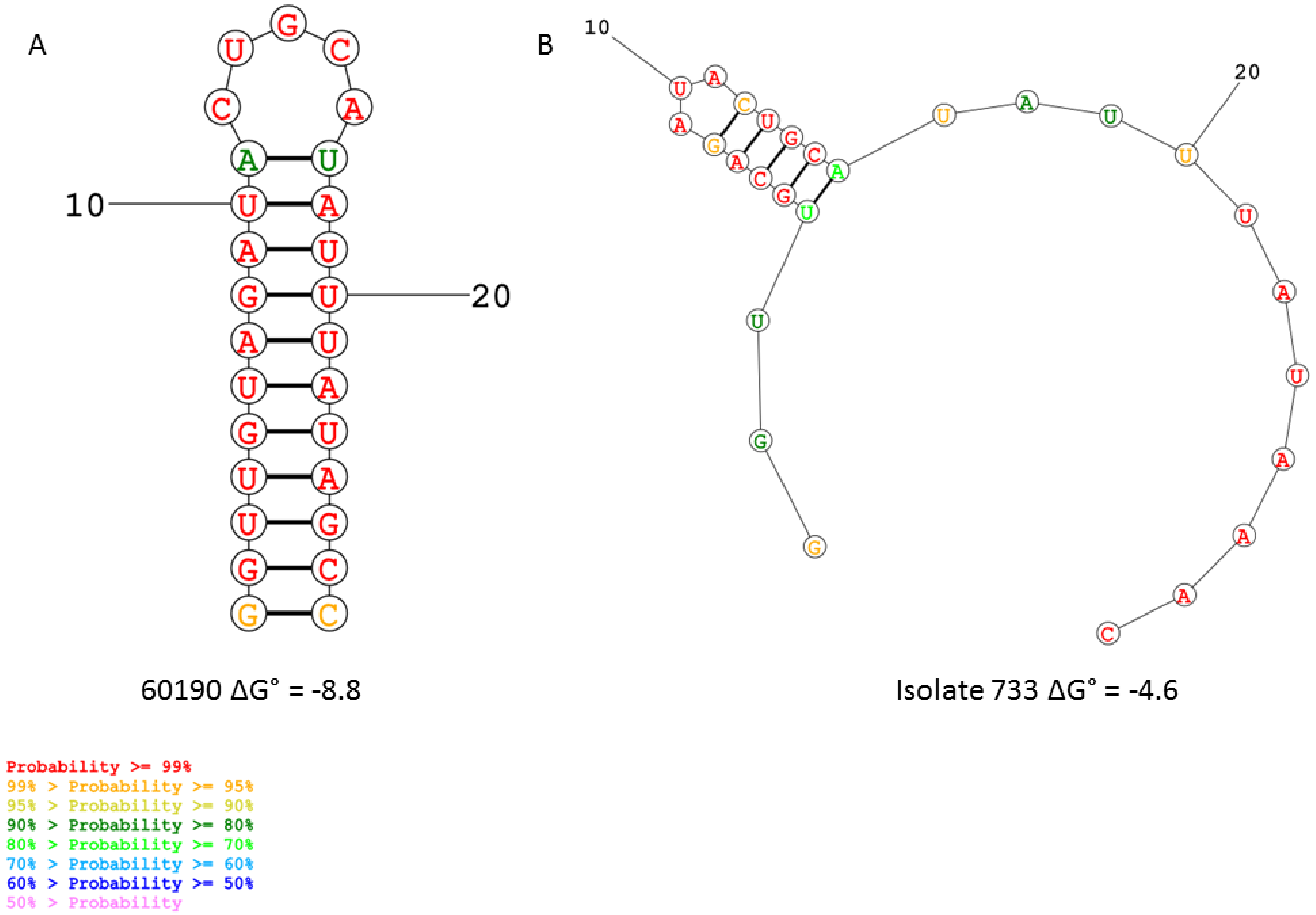

Figure S4. Predicted stem-loop structure of the *vacA* 5′ UTR in the 60190 strain and clinical isolate 733.

The nucleotide sequences of the *vacA* 5′ UTR in strain 60190 (A) and clinical isolate 733 (B), from position +4 to +30, was subjected to mRNA structure folding prediction analysis using the online MaxExpect RNAstructure Web server (Reuter & Mathews, 2010). The predicted minimum free energy values (ΔG°) for each sequence is shown.

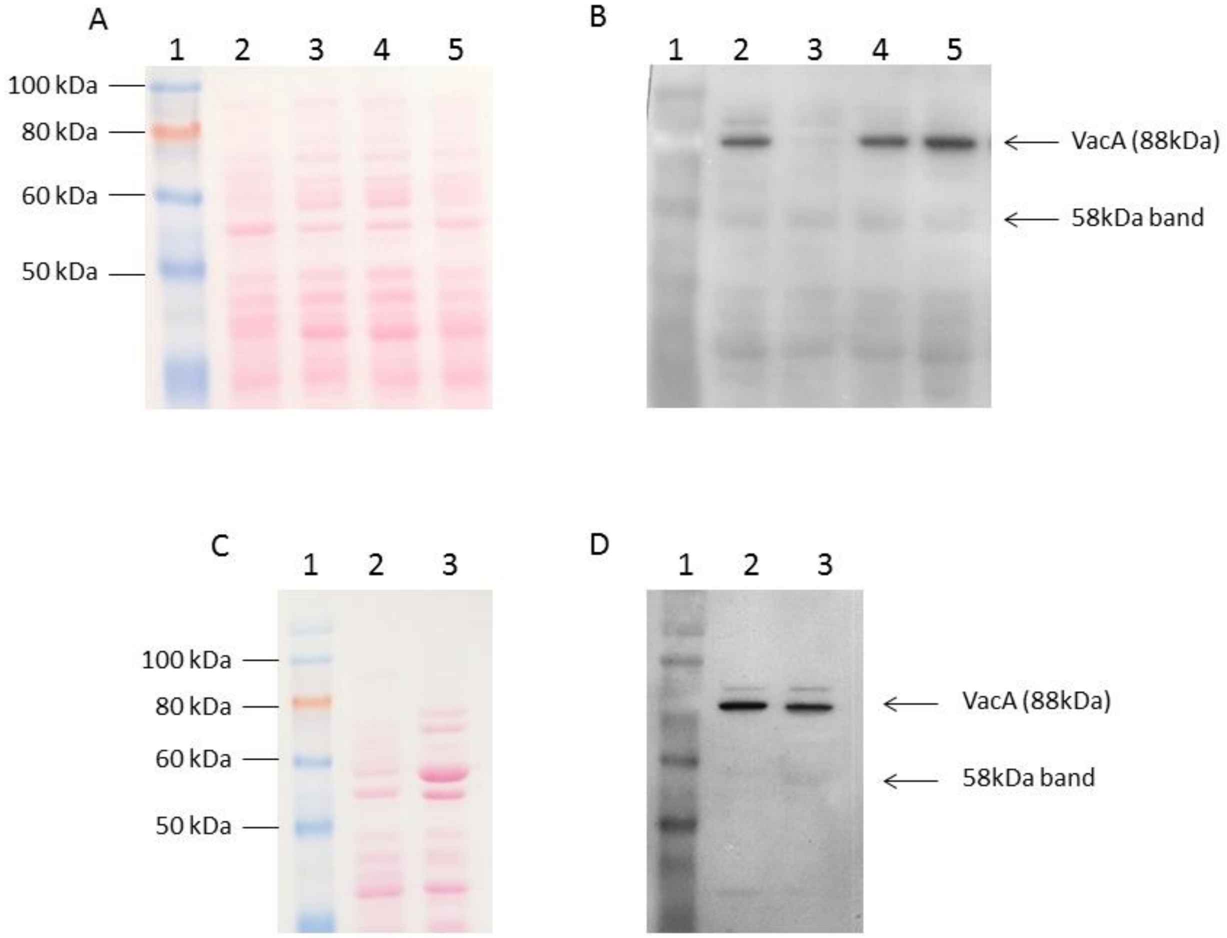

Figure S5. Protein profiles and VacA content of the 60190 wild-type strain, stem-loop mutants, and clinical *H. pylori* isolate 733 by western blotting.

(A) Nitrocellulose membrane stained with Ponceau S solution, showing protein profiles from the isogenic mutants and equal lane loading. Lane 1= Colour Plus pre-stained protein ladder (New England BioLabs), 2= 60190::pCTB2cat, 3= 60190::SLdis, 4= 60190::SLmir, 5= 60190::SLGtoA. (B) The same nitrocellulose membrane probed with a mixture of rabbit polyclonal anti-VacA p33 and p55 subunits and horseradish peroxidase-conjugated goat anti-rabbit secondary antibody, visualised using chemilluminescence. Lanes are the same as panel A. Densitometry analysis used a consistent 58 kDa non-specific band to normalise VacA quantity for gel loading. (C) Nitrocellulose membrane stained with Ponceau S solution (Sigma-Aldrich) showing protein profiles and lane loading. Lane 1 = Colour Plus pre-stained protein ladder (New England BioLabs), lane 2 = 60190, lane 3 = isolate 733. (D) Western blot probed with a mixture of rabbit polyclonal anti-VacA p33 and p55 subunits and horseradish peroxidase-conjugated goat anti-rabbit secondary antibody, visualised using chemilluminescence. Lanes are the same as panel C.

A

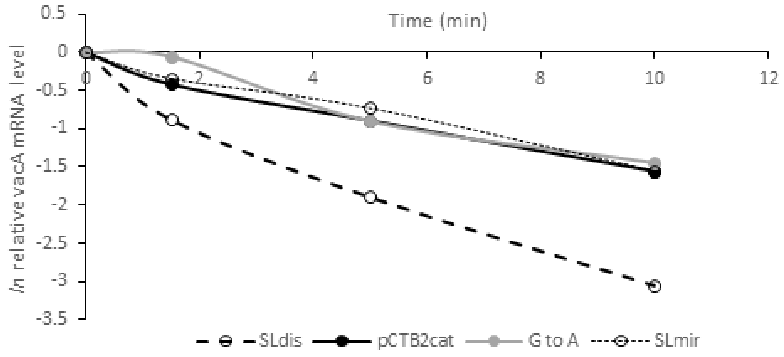

	Formula	R^2
SLdis	-0.2922x - 0.2527	0.9716
WT	-0.1489x - 0.1025	0.983
G to A	-0.1546x + 0.037	0.9641
SLmir	-0.1496x -0.0381	0.9916

B

**Strain**	**Mean mRNA half-life (SD)**	
	**Normal conditions**	**High salt conditions**
**60190::pCTB2cat**	4.7 min (0.4)	6.3 min (0.8)
**60190::SLdis**	2.9 min (0.5)	3.1 min (0.2)
**60190::SLmir**	4.8 min (0.1)	5.0 min (0.2)
**60190::G to A**	4.7 min (0.8)	ND

Figure S6. *vacA* mRNA decay curves and calculated half-life.
(A) Plot of the natural logarithm (*ln*) of relative *vacA* mRNA expression under normal salt conditions measured at each time point after rifampicin addition, determined as described in the methods section. Half-life was calculated using the formula $T_{1/2}$ mRNA= *ln*(2)/k, where *k*, the half-life co-efficient, is the slope of the curve. Straight line formulae and their fit (R^2) to the curves are given in the adjacent table.
(B) Table summarising *vacA* mRNA half-lives of SL mutants following 60 min exposure to normal and high salt culture conditions. ND = not done.
